# The quantum thermodynamic force responsible for quantum state transformation and the flow and backflow of information

**DOI:** 10.1038/s41598-019-45176-1

**Published:** 2019-06-19

**Authors:** B. Ahmadi, S. Salimi, A. S. Khorashad, F. Kheirandish

**Affiliations:** Department of Physics, University of Kurdistan, P.O. Box 66177-15175, Sanandaj, Iran

**Keywords:** Physics, Information theory and computation

## Abstract

Why do quantum evolutions occur and why do they stop at certain points? In classical thermodynamics affinity was introduced to predict in which direction an irreversible process proceeds. In this paper the quantum mechanical counterpart of the classical affinity is found. It is shown that the quantum version of affinity can predict in which direction a process evolves. A new version of the second law of thermodynamics is derived through quantum affinity for energy-incoherent state interconversion under thermal operations. we will also see that the quantum affinity can be a good candidate to be responsible, as a force, for driving the flow and backflow of information in Markovian and non-Markovian evolutions. Finally we show that the rate of quantum coherence can be interpreted as the pure quantum mechanical contribution of the total thermodynamic force and flow. Thus it is seen that, from a thermodynamic point of view, any interaction from the outside with the system or any measurement on the system may be represented by a quantum affinity.

## Introduction

In classical physics, motion is explained by the Newtonian concept of force, but what is the’driving force’ that is responsible for quantum state transformations? Why do quantum evolutions occur at all and why do they stop at certain points? From a thermodynamic point of view when a process occurs it seems like there exists a thermodynamic force which implements the change in the process. For example in the case of the free expansion of a classical gas, the gas *tends* to expand and it looks like there exists a force inside the gas which pushes the gas to expand. Therefore it is reasonable to seek the same viewpoint in quantum thermodynamics. What is the force that causes a quantum system to go from the state *ρ* to another state *σ*? What is the force that causes the energy transfer between two quantum systems in the form of heat? What is the force that causes two quantum systems to establish correlations? And what is the force that causes the flow and backflow of information in quantum evolutions? In classical thermodynamics, chemists proposed the same question concerning chemical reactions. Chemists called the force that caused chemical reactions *affinity*. The thermodynamic formulation of affinity as we know it today is due to Théophile De Donder (1872–1957), the founder of the Belgian school of thermodynamics. He formulated chemical affinity on the basis of chemical potential^1^. For a chemical reaction $X+Y\rightleftharpoons 2Z$, he defined a new state variable called affinity as^2^

1 $$A\equiv {\mu }_{X}+{\mu }_{Y}-2{\mu }_{Z},$$

where the coefficients *μ*_*k*_ are called the chemical potentials. This affinity is the driving force for chemical reactions. In terms of affinity *A*, the rate of increase of entropy production, *d*_*i*_*S*, is written as^2^

2 $$\frac{{d}_{i}S}{dt}=(\frac{A}{T})\frac{d\xi }{dt}.$$

Thus the entropy production due to chemical reactions is a product of a thermodynamic force *A*/*T* and a thermodynamic flow *dξ*/*dt*. The flow in this case is the conversion of reactants to products (or vice versa), which is caused by the force *A*/*T*. The thermodynamic flow *dξ*/*dt* is referred to as the velocity of reaction or rate of conversion. there is no general relationship between the affinity and the velocity of a reaction. The sign of affinity can be used to predict the direction of reaction. If *A* > 0, the reaction proceeds to the right and if *A* < 0, the reaction proceeds to the left^2^.

In classical thermodynamics, the affinity is the *tendency* of the system to evolve in a particular direction. Therefore the main aim of our work is to find the quantum mechanical counterpart of this classical affinity and ensure that it is the tendency of the quantum system to evolve in a specific direction. In order to this we will show that, as in classical thermodynamics, in quantum thermodynamics the entropy production of a system can be expressed as the product of a thermodynamic force and a thermodynamic flow. We then ensure that the quantum affinity, like classical affinity, acts as a force which pushes an initial state to a final state thus determining in which direction a quantum process proceeds, i.e, the quantum affinity is the tendency of a system to go from a state *ρ* to another state *σ* under a quantum mechanical evolution. This means that the quantum affinity connects the arrow of time with quantum state transformation. We will also show that the quantum mechanical affinity can be considered as a good candidate to be responsible for the flow and backflow of information in Markovian and non-Markovian evolutions. Hence the quantum affinity connects also the arrow of time with the flow and backflow of information. We will finally show that the rate of the quantum coherence is the difference between the total quantum thermodynamic force and flow and the classical thermodynamic force and flow, as expected.

## Results

### Quantum thermodynamic force and flow

Consider an arbitrary quantum system *S* coupled with a heat reservoir *B* initially in thermal state at temperature *T*. The total system *S* + *B* with Hamiltonian *H* = *H*_*S*_ + *H*_*B*_ + *H*_*SB*_ is closed and thus evolves unitarily in time^3,4^. But we are primarily interested in the occurrence and characterization of irreversible behavior in the system. We thus focus our attention on the entropy *S*(*t*) of the system^4^, *S*(*t*) = −*tr*{*ρ*_*s*_(*t*)ln*ρ*_*s*_(*t*)}, where *ρ*_*s*_(*t*) is the density of the state of the system which evolves from an initial state *ρ*_*s*_(0) to a final state *ρ*_*s*_(*t*) by completely positive and trace preserving (CPTP) maps Λ_*t*_^3^, i.e, *ρ*_*s*_(*t*) = Λ_*t*_[*ρ*_*s*_(0)]. The total change in the entropy Δ*S* of the system is divided into two parts^2,5^

3 $${\rm{\Delta }}S={{\rm{\Delta }}}_{i}S+{{\rm{\Delta }}}_{e}S,$$

in which Δ_*e*_*S* is the entropy change due to the exchange of matter and energy with the environment and Δ_*i*_*S* the entropy change due to “uncompensated transformation”, the entropy produced by the irreversible processes in the interior of the system. Δ_*e*_*S* equals $\frac{\langle Q\rangle }{T}$ where 〈*Q*〉 ≡ *tr*{Δ*ρH*} is the heat exchanged between the system and the reservoir. Thus substituting *S*(*ρ*) = −*tr*{*ρ*_*s*_(*t*)ln*ρ*_*s*_(*t*)} into Eq. (3) then taking the time derivative of Eq. (3), after some straightforward calculations (see Supplementary Note 1 for more details), we get

4 $$\frac{{d}_{i}S}{dt}=tr\{({\dot{\rho }}_{s}(t){\rho }_{s}^{\beta })(\frac{1}{{\rho }_{s}^{\beta }}(\mathrm{ln}\,{\rho }_{s}^{\beta }-\,\mathrm{ln}\,{\rho }_{s}(t)))\}.$$

Now, analogous to De Donder’s definition, we define the thermodynamic force and flow, respectively, as

5 $${F}_{th}=\frac{1}{{\rho }_{s}^{\beta }}(\mathrm{ln}\,{\rho }_{s}^{\beta }-\,\mathrm{ln}\,{\rho }_{s}(t)),$$

6 $${V}_{th}={\dot{\rho }}_{s}(t){\rho }_{s}^{\beta }.$$

Thus, as in the case of the entropy production due to chemical reactions in classical thermodynamics, the entropy production due to irreversible processes in quantum thermodynamics is written as a product of a thermodynamic force $\frac{1}{{\rho }_{s}^{\beta }}(\mathrm{ln}\,{\rho }_{s}^{\beta }-\,\mathrm{ln}\,{\rho }_{s}(t))$ and a thermodynamic flow ${\dot{\rho }}_{s}(t){\rho }_{s}^{\beta }$. The flow in this case is the transformation of an initial quantum state to a final state. In ref.^6^ $\dot{V}$ was introduced to describe the speed of the system evolution, where *V* = *tr*{*ρρ*_*s*_} and *ρ*_*s*_ is the density matrix of the target state |*S*〉. Notice that if the system Hamiltonian is time-dependent ${\rho }_{s}^{\beta }$ is replaced by ${\rho }_{s}^{\beta }(t)=\exp (\,\,-\,\beta {H}_{s}(t))/{Z}_{s}(t)$. It must also be noted that in our work ${\rho }_{s}^{\beta }(t)$ is not the target state. It is the instantaneous equilibrium state of the system corresponding to the bath temperature *T*. What is important about ${\rho }_{s}^{\beta }(t)$ in our work is that the quantum thermodynamic force vanishes in this state and it remains zero if the evolution is Markovian. But if the evolution is non-Markovian it may not remain zero because the Gibbs state may not be an invariant state of the non-Markovian map^7^. Now comparing to Eq. (2), ${\rho }_{s}^{\beta }$ and $\mathrm{ln}\,{\rho }_{s}^{\beta }-\,\mathrm{ln}\,{\rho }_{s}(t)$ play the roles of the temperature *T* and the affinity *A*, respectively. Therefore *F*_*th*_ can be rewritten as

7 $${F}_{th}=\frac{A}{{\rho }_{s}^{\beta }}.$$

It should be mentioned that it is a bit misleading to state that ${\rho }_{s}^{\beta }$ plays the role of the temperature. We have just added ${\rho }_{s}^{\beta }$ to Eq. (4) to define quantum affinity in Eq. (5), in analogues to De Donder’s definition of affinity, and adding ${\rho }_{s}^{\beta }$ does not change any results. From now on, we shall refer to *A* as the *quantum* affinity and the thermodynamic flow will be referred to as the velocity of the transformation. Here we define $\bar{A}$ as

8 $$\bar{A}(\rho )\equiv tr\{A(\rho )\}.$$

In the following we will show that, as in classical thermodynamics, the quantum affinity *A* acts as a force and determines the direction in which the quantum processes proceed.

#### Pure bipartite states

Here we examine the transformation of pure bipartite states under local operations and classical communication (LOCC). First we must point out that in this particular case no bath (and temperature) is present and consequently no heat is exchanged thus ${\rho }_{s}^{\beta }$ does not appear then the quantum affinity becomes −ln*ρ*_*s*_(*t*). A quantum state *ρ* can be transformed into another quantum state *σ* by LOCC (see Supplementary Note 2) if and only if

9 $$\bar{A}(\rho )\le \bar{A}(\sigma ).$$

Equation (9) shows that $\bar{A}$(*ρ*) is the tendency of the state *ρ* to go to the state *σ*. As in classical thermodynamics that affinity was expressed on the concept of chemical potential, $\bar{A}$(*ρ*) can be interpreted as the average local non-equilibrium potential of the state *ρ*. In other words a state *ρ* with a smaller potential is “pulled”, by LOCC, toward the state *σ* with a larger potential. Thus the quantum affinity *A*(*ρ*) associates each state of the system with a local potential that determines whether a state can be (deterministically) transformed into another state by LOCC. We must point out that our definition of the quantum affinity is also valid for matrices with zero eigenvalues. When the eigenvalue of a density matrix goes to zero the affinity becomes larger and approaches infinity. Thus the affinity of a density matrix with a zero eigenvalue is infinity and the above statement still holds. For example if a pure bipartite entangled quantum state *ρ* has more zero eigenvalues than another pure bipartite entangled quantum state *σ* then $\bar{A}(\rho ) > \bar{A}(\sigma )$. Thus the state *σ* can be transformed with certainty to the state *ρ* by LOCC which completely agrees with Nielsen’s Theorem^8^. Hatano and Sasa^9^ introduced a classical non-equilibrium potential as *ϕ*(*x*; *α*) = −ln*ρ*_*ss*_(*x*; *α*) where *ρ*_*ss*_(*x*; *α*) is the probability distribution function of the steady state corresponding to *α*. Similarly, Manzano *et al*.^10^ defined Φ_*ρ*_ = −ln*ρ* as the quantum non-equilibrium potential. Thus our work justifies the definition of the speed of the quantum system evolution introduced in ref.^6^ and the definition of the non-equilibrium potential of the quantum system introduced in refs^9,10^.

There exist, however, incomparable states in the sense that neither state is convertible into the other with certainty only using LOCC. Let |*ψ*〉 and |*ϕ*〉 be two states with Schmidt numbers *α* and *β*, respectively. The transformation |*ϕ*〉 → |*ψ*〉 is more probable than |*ψ*〉 → |*ϕ*〉 by LOCC (see Supplementary Note 3) if and only if the $\ell$-th component of the potential difference

10 $${\rm{\Delta }}{A}_{\ell }={A}_{\ell }({\rho }_{\psi })-{A}_{\ell }({\rho }_{\varphi }) > 0.$$

### Quantum affinity and the second law

A state *ρ* block diagonal in the energy eigenbasis can be transformed with certainty into another block diagonal state *σ* by thermal operations (see Supplementary Note 4) if and only if

11 $$\bar{A}(\hat{\alpha }) > \bar{A}(\hat{\beta }),$$

in which *α*, *β* are the probability vectors of the states *ρ* and *σ*, respectively. Equation (11) is another way of stating the second law of thermodynamics for states block diagonal in energy: “$\bar{A}(\hat{\alpha })$ of a state *ρ* with probability vector *α* never increases under thermal operations”. Hence, the quantum affinity *A*(*ρ*) connects the arrow of time with quantum state transformation. In other words, the thermodynamic arrow of time always points in the direction of decreasing the quantum state affinity $\bar{A}(\hat{\alpha })$ under thermal operations.

#### Quantum affinity, heat and work

The rate of the entropy production of a quantum system interacting with a reservoir initially in equilibrium at temperature *T* can be written as

12 $$\frac{{d}_{i}S}{dt}=tr\{{\dot{\rho }}_{s}{A}^{tot}\}-tr\{{\dot{\rho }}_{s}{A}^{eq}\},$$

where the total quantum affinity was defined as *A*^*tot*^ ≡ −ln*ρ*_*s*_(*t*) and the equilibrium quantum affinity as ${A}^{eq}\equiv -\,\mathrm{ln}\,{\rho }_{s}^{\beta }$. Thus the (irreversible) quantum affinity can be expressed as the difference between the total and the equilibrium quantum affinity

13 $$A(t)={A}^{tot}-{A}^{eq}.$$

Using Eq. (13) the heat could be expressed as

14 $$d\langle Q\rangle =tr\{\rho (t+dt){\mathbb{Q}}\}-tr\{\rho (t){\mathbb{Q}}\},$$

where ℚ = −*TA*^*eq*^. Hence the equilibrium quantum affinity is the force which pushes (pulls) information out of (into) the system to (from) its environment in the form of heat. Now the (irreversible) quantum affinity can be interpreted as the force which is responsible for the information exchanged, between the system and the environment, not in the form of heat. This type of information exchange occurs in the interior of the system and thus may be reused by the system to do work. For instance information may be stored in the *correlations* established during the strong interaction of the system with its environment. In the next section we will show that whenever $\bar{A}(\rho )$ begins to increase information backflows into the system. Consider the free expansion of an isolated (classical) gas of non-interacting particles. It seems like there exists a force which pushes the gas to expand (or to become more disordered). The relation *d*_*i*_*S* = *F*_*th*_*dξ*, in classical thermodynamics, is similar to the relation, in classical mechanics, *dW* = *Fdx*. *F*_*th*_ and *dξ* play the roles of the force *F* and the displacement *dx*, respectively. *d*_*i*_*S* is in fact equal to *βdW*_*irr*_ where *dW*_*irr*_ is the irreversible work and is always positive in (deterministic) classical thermodynamics due to the Clausius’ statement of the Second Law, thus information is always encoded which, in turn, leads to the fact that the Carnot engine is the most efficient engine. In a further publication^11^ we will examine more properties and uses of the quantum affinity in quantum thermodynamics as a force. We will show that the relation *d*_*i*_*S* = *βdW*_*irr*_ also holds in quantum thermodynamics and the quantum affinity is responsible for encoding and decoding information. Whenever it decodes information more work, than what is expected, can be extracted from the system leading to an engine more efficient than that of Carnot. It will also be shown that Maxwell’s demon^12^ in quantum thermodynamics is in fact a quantum affinity which forces the information back into the system, i.e., it decodes information. We will also reestablish the Landaure’s principle^13^ in the language of the quantum affinity. It is worth mentioning that, from a thermodynamic point of view, LOCC are in fact Maxwell’s demons intervening in the process and inequalities (9) and (10) mean that it seems like there exists a force implementing the change LOCC make on the state of the system. The details mentioned above indicate the fact that, from a thermodynamic point of view, any interaction from the outside with the system or any measurement on the system may be represented by a quantum affinity.

### Quantum affinity and non-Markovianity

During a Markovian evolution information flows out of the system into its environment but in a non-Markovian evolution, due to correlations between the system and its environment, information backflows into the system from its environment^14^. Here we will show that the quantum affinity is the tendency of the system to establish correlations with its environment and is the driving force responsible for the flow and backflow of information. Hence the quantum affinity connects the arrow of time with the flow and backflow of information. The quantum affinity *A*(*ρ*_*s*_(*t*)) is a function of the map Λ_*t*_, thus it behaves differently during the flow and the backflow. The collapses (revivals) of *A*(*ρ*_*s*_(*t*)) in the first example, below, indicate the fact that the information flows out of (backflows into) the system during the evolution. But it should be noted that *A*(*ρ*_*s*_(*t*)) is the total thermodynamic affinity at time t. Consider a master equation with two decay rates, *γ*_1_(*t*) > 0 and *γ*_2_(*t*) < 0, which means that both the flow and the backflow of information occurs during the evolution of the system. If the flow dominates the backflow, i.e, |*γ*_1_(*t*)| > |*γ*_2_(*t*)| then revivals are not observed in the behavior of $\bar{A}({\rho }_{s}(t))$, although there exists backflow of information in the dynamics at all times (see the second example). In order to separate the contributions of the flow and the backflow in $\bar{A}({\rho }_{s}(t))$ we take the time derivative of $\bar{A}({\rho }_{s}(t))$ (see Supplementary Note 5)

15 $$\frac{d\bar{A}({\rho }_{s}(t))}{dt}=\sum _{k=1}^{{d}^{2}-1}\,\frac{d{\bar{A}}^{k}({\rho }_{s}(t))}{dt},$$

where

16 $$\frac{d{\bar{A}}^{k}}{dt}\equiv -\,tr\{{\gamma }_{k}(t)[{L}_{k}(t){\rho }_{s}{L}_{k}^{\dagger }(t)-\frac{1}{2}\{{L}_{k}^{\dagger }(t){L}_{k}(t),{\rho }_{s}\}]{\rho }_{s}^{-1}\}.$$

Now the effect of the tendency for the flow and backflow of information can be clearly seen separately. The tendency for the flow of information is the tendency of the system to lose information and the tendency for the backflow of information is the tendency of the system to regain information. In the following examples we will illustrate how $\bar{A}({\rho }_{s}(t))$ behaves differently when the system tends to lose information and regain information during the dynamics and we see that if *γ*_*k*_ = _*i*_(*t*) > 0 then *A*^*i*^ is the force driving the flow of information and if *γ*_*k*_ = _*j*_(*t*) < 0 then *A*^*j*^ is the force driving the backflow of information.

#### Examples

**Figure 1:**
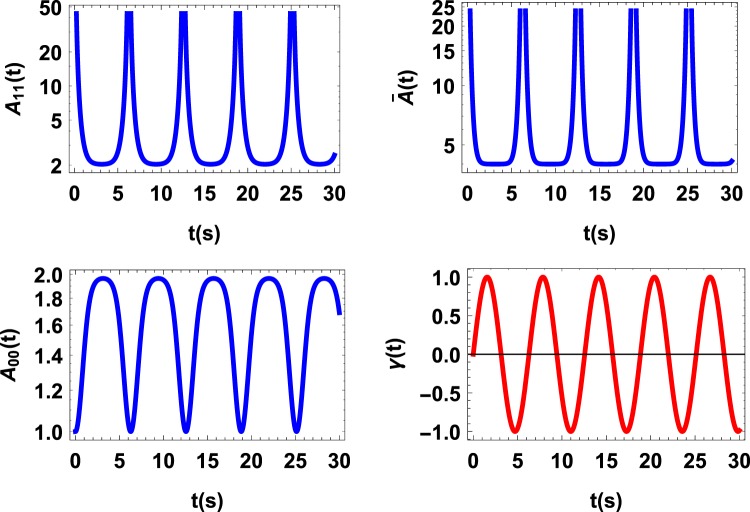
*A*(*ρ*_*s*_(*t*)) vs. time t for a qubit with decay rate *γ*(*t*) = sin(*t*). As is anticipated revivals and collapses are observed that give rise to the temporary flow and backflow of information which suggest that *A*(*ρ*_*s*_(*t*)) is the force responsible for driving the flow and backflow of information.

Since $\mathrm{ln}\,{\rho }_{s}^{\beta }$ does not change over the time we neglect this term when we calculate the quantum affinity *A*(*ρ*_*s*_(*t*)) for these examples. The following dynamical map of a two-dimensional quantum system (qubit),

17 $${\dot{\rho }}_{s}(t)=\gamma (t)[{\sigma }_{z}{\rho }_{s}(t){\sigma }_{z}-{\rho }_{s}(t)],$$

where *σ*_*z*_ is the Pauli matrix and

18 $$\gamma (t)=\,\sin (t),$$

with ${\int }_{{t}_{0}}^{{t}_{1}}\gamma (s)ds\ge 0$ for completely positive dynamics, is of particular interest, as it provides a simple example of a completely positive evolution^15^ which causes the system to lose information when *γ*(*t*) is positive and to regain information when *γ*(*t*) is negative. As can be seen from the results plotted in Fig. (1) for the initial state $${\rho }_{s}(0)=\frac{1}{2}(\begin{array}{ll}1 & 1\\ 1 & 1\end{array})$$, when the system changes its tendency from losing information (the flow) to regaining information (the backflow) the rate of *A*_*ii*_(*ρ*_*s*_(*t*)) begins to switch signs. It is also observed that during the evolution $\bar{A}({\rho }_{s}(t))$ decreases and increases giving rise to the temporary flow and backflow of information.

**Figure 2:**
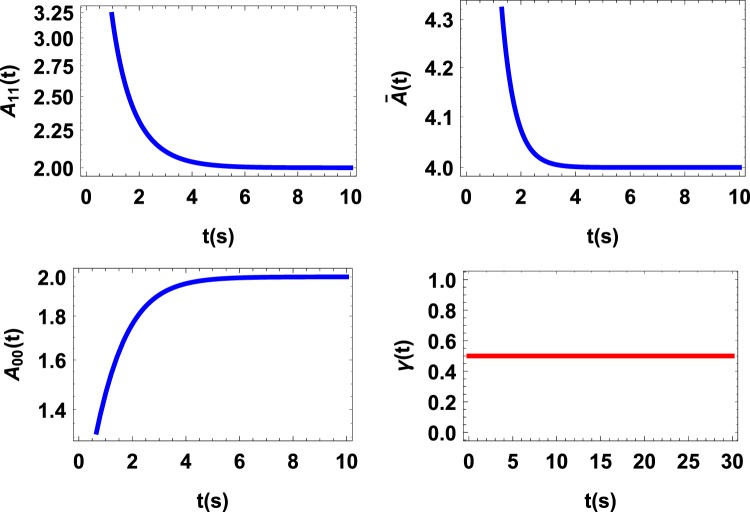
*A*(*ρ*_*s*_(*t*)) vs. time t for purely dephasing dynamics with decay rate *γ*(*t*) = 1/2. Since no backflow of information occurs no revival or collapse is observed.

As a second example consider the evolution of a qubit given by the following master equation (pure dephasing)^3^,

19 $${\dot{\rho }}_{s}(t)=\gamma (t)[{\sigma }_{z}{\rho }_{s}(t){\sigma }_{z}-{\rho }_{s}(t)],$$

where *σ*_*z*_ is the Pauli matrix and *γ*(*t*) = 1/2, therefore only the flow of information occurs. For the initial state $${\rho }_{s}(0)=\frac{1}{2}(\begin{array}{ll}1 & 1\\ 1 & 1\end{array})$$, a straightforward computation of quantum affinity gives that, as expected, there exist no revival and collapse in the behavior of *A*_*ii*_(*ρ*_*s*_(*t*)) and consequently no revival and collapse in $\bar{A}({\rho }_{s}(t))$ (see illustration in Fig. 2). Since no backflow of information occurs, $\bar{A}({\rho }_{s}(t))$ decreases (collapses) with time and never increases (revives).

We finally consider an example of multiply decohering dynamics^16,17^,

20 $${\dot{\rho }}_{s}(t)=\frac{1}{2}\sum _{k=1}^{3}\,{\gamma }_{k}(t)[{\sigma }_{k}{\rho }_{s}(t){\sigma }_{k}-{\rho }_{s}(t)],$$

where the *σ*_*k*_ are the Pauli *σ* matrices and

21 $${\gamma }_{1}(t)={\gamma }_{2}(t)=1,\,{\gamma }_{3}(t)=-\,\tanh \,t.$$

**Figure 3:**
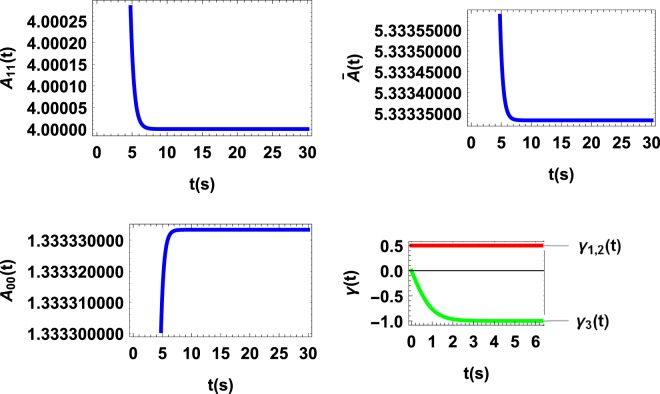
*A*(*ρ*_*s*_(*t*))vs. time t for multiply decohering dynamics with decay rates γ1(t) = γ2(t) = 1 and γ_3_(t) = −tanh *t*. Although information backflows into the system at all times but since the first two flow forces dominate the backflow one no revival and collapse may appear.

**Figure 4:**
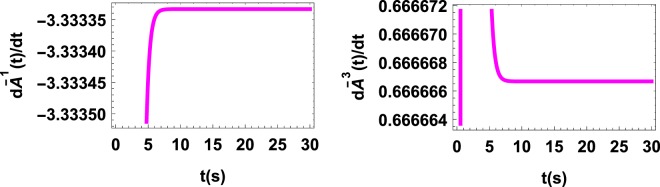
$\frac{d{\bar{A}}^{1}}{dt}$ is negative, but $\frac{d{\bar{A}}^{3}}{dt}$ is positive at all times showing the fact that revival (backflow) of information occurs throughout the evolution, although no revival or collapse is observed in the behavior of *A*(*t*) (see Fig. 3).

As mentioned before, the fact that *A*_*ii*_(*ρ*_*s*_(*t*)) undergoes no collapse and revival with time (as shown in Fig. 3) is because the first two flow forces dominate the backflow one. As depicted in Fig. (4), for the initial state $${\rho }_{s}(0)=\frac{1}{2}(\begin{array}{ll}1 & 1\\ 1 & 1\end{array})$$, $\frac{d{\bar{A}}^{1}}{dt}$ and $\frac{d{\bar{A}}^{2}}{dt}$ are negative but $\frac{d{\bar{A}}^{3}}{dt}$ is positive, during the entire evolution, which indicates that *A*^3^ is responsible for the backflow of information. Considering these results it stands to reason to interpret the quantum affinity *A*(*ρ*_*s*_(*t*)) as a thermodynamic force driving the flow and backflow of information or the tendency of the system to establish correlations with its environment.

### Quantum coherence and quantum thermodynamic force and flow

Quantum coherence is a landmark feature of quantum mechanics and has no classical counterpart. It has no classical counterpart because it comes from the off-diagonal elements of the density matrix and is also basis-dependent. Similar to entanglement, even more fundamental, coherence can be regarded as a resource^18,19^. Although it has been recently shown that quantum coherence plays a role in transitions under thermal operations^19–25^ we still lack a complete and satisfactory understanding of the role of quantum coherence in thermodynamics. Here we separate the classical contribution of the total thermodynamic force and flow and show that the difference between the total and classical thermodynamic force and flow equals the rate of quantum coherence. By classical, here, we mean those elements of the density matrix which generate no quantum coherence in the state of the system. In order to derive a relation between quantum coherence and quantum thermodynamic force and flow we will employ the so-called relative entropy of coherence^19,26^

22 $$C(\rho )=S({\rho }_{d})-S(\rho ),$$

where *ρ*_*d*_ is the state obtained from *ρ* by deleting all the off-diagonal elements. Now let {|*n*〉} denote the eigenstates of the Hamiltonian *H*, and *p*_*n*_ = 〈*n*|*ρ*|*n*〉 the corresponding populations. Thus we fix the eigenbasis to be the energy eigenbasis. After some straightforward calculations the entropy production can be written as^20,21^

23 $$\frac{{d}_{i}S}{dt}=\sum _{n}\,{\dot{p}}_{n}\,\mathrm{ln}\,\frac{{p}_{n}^{\beta }}{{p}_{n}}-\dot{C}({\rho }_{s}).$$

Thus the desired relation is

24 $$-\dot{C}({\rho }_{s})=tr\{{\dot{\rho }}_{s}\,\mathrm{ln}\,\frac{{\rho }_{s}^{\beta }}{{\rho }_{s}}\}-\sum _{n}\,{\dot{p}}_{n}\,\mathrm{ln}\,\frac{{p}_{n}^{\beta }}{{p}_{n}},$$

where the first term on the right hand side is the total thermodynamic force and flow and the second term is the classical part of the total thermodynamic force and flow. Equation (24) means that $-\dot{C}({\rho }_{s})$ is obtained by subtracting the classical part from the total thermodynamic force and flow. Therefore what remains is *purely* quantum mechanical. This result is remarkable, because we have shown that, in the language of thermodynamic force and flow, the rate of quantum coherence can be interpreted as the pure quantum mechanical contribution of the total thermodynamic force and flow. Roughly speaking, $-\dot{C}({\rho }_{s})$ is the off-diagonal contribution of the total thermodynamic force and flow. Therefore we have shown that in cases like the flow and backflow of information and quantum coherence which are specific features of (stochastic) quantum mechanics the quantum affinity still acts as a force or tendency.

## Conclusions

We have shown that, as in classical thermodynamics, the entropy production can be written as the product of a thermodynamic force and a thermodynamic flow. The latter determines the velocity of the evolution. Comparing quantum thermodynamic force with its classical version we have derived the quantum mechanical version of affinity and proved that, as in classical thermodynamics, quantum affinity can predict in which direction an irreversible transformation occurs. This quantum affinity enabled us to associate a state with a local non-equilibrium potential such that pure bipartite entangled quantum states with smaller potentials are pulled toward pure bipartite entangled states with larger potentials, deterministically or nondeterministically, under LOCC. We have examined the behavior of quantum affinity under thermal operations and discovered a new version of the Second Law through quantum affinity such that the thermodynamic arrow of time always points in the direction of decreasing quantum affinity. we have also observed that quantum affinity can be interpreted as the thermodynamic force driving the flow and backflow of information in Markovian and non-Markovian evolutions and illustrated this with three physical examples. And lastly, using the concept of relative entropy of coherence, we have shown that in the language of thermodynamic force and flow the rate of quantum coherence can be interpreted as the pure quantum mechanical contribution of the total thermodynamic force and flow. Thus we have shown that, from a thermodynamic point of view, any interaction from the outside with the system or any measurement on the system may be represented by a quantum affinity^27–33^.

## Supplementary information

Supplementary
