# Supplementary material for: The quantum thermodynamic force responsible for quantum state transformation and the flow and backflow of information
Source: Sci Rep. 2019 Jun 19;9:8746. doi: 10.1038/s41598-019-45176-1 (PMC6584574; doi:10.1038/s41598-019-45176-1)
Supplement: Supplementary file 1 — Supplementary [file 41598_2019_45176_MOESM1_ESM.pdf]

The quantum thermodynamic force responsible for quantum state transformation and the flow and backflow of information

B. Ahmadi[1], **S. Salimi**[1,*], **A. S. Khorashad**[1], **and F. Kheirandish**[1]

[1]Department of Physics, University of Kurdistan, P.O.Box 66177-15175, Sanandaj, Iran
[*]Correspondence and requests for materials should be addressed to S. Salimi. (email: shsalimi@uok.ac.ir)

1 Supplementary Note 1

Clausius considered irreversible processes as an integral part of formulating the second law of thermodynamics. He included irreversible processes explicitly into the formalism of entropy by dividing entropy into two parts[2]: the change in entropy due to the exchange of heat with the environment by the term $\Delta Q/T$ (which is compensated by equal gain or loss of heat by the environment) and the entropy produced by irreversible processes within the system (the uncompensated transformation) d_iS. Thus the total change in the entropy ΔS of the system is divided into two parts[2,5]

$$\Delta S = \Delta_i S + \Delta_e S, \tag{1}$$

in which $\Delta_e S$ is the entropy change due to the exchange of matter and energy with the environment and $\Delta_i S$ the entropy change due to "uncompensated transformation", the entropy produced by the irreversible processes in the interior of the system. $\Delta_e S$ equals $\frac{\langle Q\rangle}{T}$ where $\langle Q\rangle$ is the heat exchanged between the system and the reservoir[2,5]. For any quantum dynamical process with $\dim(\mathcal{H}) < +\infty$, the rate of the entropy change is given by[27]

$$\frac{dS}{dt} = -tr\{\dot{\rho}_s(t)\ln\rho_s(t)\}.$$

Thus substituting $S(\rho) = -tr\{\rho_s(t)\ln\rho_s(t)\}$ into Eq. (1) then taking the time derivative of Eq. (1) we have

$$-tr\{\dot{\rho}_s(t)\ln\rho_s(t)\} = \frac{d_iS}{dt} + \frac{\langle\dot{Q}\rangle}{T}, \tag{2}$$

where[28]

$$\langle\dot{Q}\rangle \equiv tr\{\dot{\rho}_s(t)H_s\}. \tag{3}$$

After some straightforward calculations we get[29]

$$\frac{d_iS}{dt} = tr\{(\dot{\rho}_s(t)\rho_s^{\beta})(\frac{1}{\rho_s^{\beta}}(\ln\rho_s^{\beta} - \ln\rho_s(t)))\}, \tag{4}$$

where $\rho_s^{\beta} = \exp(-\beta H_s)/Z_s$ is the Gibbs state of the system. Now, analogous to De Donder's definition, we define the thermodynamic force and flow, respectively, as

$$F_{th} \equiv \frac{1}{\rho_s^{\beta}}(\ln\rho_s^{\beta} - \ln\rho_s(t)), \tag{5}$$

$$V_{th} \equiv \dot{\rho}_s(t)\rho_s^{\beta}. \tag{6}$$

2 Supplementary Note 2

Nielsen proved[8] that a pure bipartite entangled quantum state $|\psi\rangle$ can be transformed into another pure bipartite entangled state $|\phi\rangle$ by local operations and classical communication (LOCC) if and only if $\alpha \prec \beta$, where the probability vectors α and β denote the Schmidt coefficient vectors of $|\psi\rangle$ and $|\phi\rangle$, respectively. Here the symbol $\prec$ stands for the "majorization". We refer the reader to read Ref.[8] and the references cited therein to read more about LOCC. We denote a quantum state by the probability vector of its Schmidt coefficients. An n-dimensional probability vector x is said to be majorized by another n-dimensional probability vector y, written $x \prec y$, if the following relation holds:

$$\sum_{i=1}^{\ell} x_i^{\downarrow} \leq \sum_{i=1}^{\ell} y_i^{\downarrow} \quad \text{for any} \quad 1 \leq \ell < n, \tag{7}$$

where $x^{\downarrow}$ denotes the vector obtained by sorting the components of x in nonincreasing order.

Theorem 1 *(Theorem II.3.1 of Ref.[30]). Let $x, y \in \mathbb{R}^n$. Then the following two conditions are equivalent:*
(i) $x \prec y$.
(ii) $tr\varphi(x) \leq tr\varphi(y)$,
for all convex functions φ from $\mathbb{R}$ to $\mathbb{R}$, where $tr\varphi(x) \equiv \sum_{i=1}^{n}\varphi(x_i)$.

Since $\bar{A}$ is a convex function, using this theorem and Nielsen's theorem[8], we conclude that the state ρ can be transformed into the state σ by LOCC if and only if $\bar{A}(\rho) \leq \bar{A}(\sigma)$.

3 Supplementary Note 3

Vidal[31] discovered that there is always a maximal probability for incomparable states to be transformed into each other. Let $P(|\psi\rangle \to |\phi\rangle)$ denote the maximal transformation probability of obtaining the state $|\phi\rangle$ from $|\psi\rangle$ by LOCC, then

$$P(|\psi\rangle \to |\phi\rangle) = \min_{1\le\ell\le n} \frac{E_\ell(\alpha)}{E_\ell(\beta)} = \frac{\alpha_n + \alpha_{n-1} + ... + \alpha_\ell}{\beta_n + \beta_{n-1} + ... + \beta_\ell}. \tag{8}$$

where n is the maximum of the Schmidt coefficients of $|\psi\rangle$ and $|\phi\rangle$, and $E_\ell(x)$ denotes the abbreviation of $\sum_{i=\ell}^{n} x_i^{\downarrow}$ for probability vector x. In the following we prove a theorem to show that the potential difference between two states predicts which state is more probable to be transformed (or pulled) into another.

Theorem 2 *Let $|\psi\rangle$ and $|\phi\rangle$ be two states with Schmidt numbers α and β, respectively. The ℓ-th component of the potential difference $\triangle A_\ell = A_\ell(\rho_\psi) - A_\ell(\rho_\phi) > 0$ if and only if the transformation $|\phi\rangle \to |\psi\rangle$ is more probable than $|\psi\rangle \to |\phi\rangle$ by LOCC.*

Proof. Consider the two states $|\psi\rangle$ and $|\phi\rangle$ with Schmidt numbers α and β, respectively. Thus the potential difference $\triangle A$ between these states reads,

$$A(\rho_\psi) - A(\rho_\phi) = (\ln\frac{\beta_1}{\alpha_1}, \ln\frac{\beta_2}{\alpha_2}, ..., \ln\frac{\beta_\ell}{\alpha_\ell}, ..., \ln\frac{\beta_n}{\alpha_n}).$$

Suppose there exist some α_j, β_j such that $\frac{\alpha_j}{\beta_j} < \frac{\alpha_\ell}{\beta_\ell} < 1$ for $j \ne \ell$. Thus $\frac{\alpha_\ell}{\alpha_j} > \frac{\beta_\ell}{\beta_j}$. Rewriting Eq. (8)

$$P(|\psi\rangle \to |\phi\rangle) = \frac{\alpha_n + \alpha_{n-1} + ... + \alpha_j + \alpha_\ell}{\beta_n + \beta_{n-1} + ... + \beta_j + \beta_\ell},$$

and since $\frac{\alpha_\ell}{\alpha_j} > \frac{\beta_\ell}{\beta_j}$, we must have

$$\frac{\alpha_n + \alpha_{n-1} + ... + \alpha_j}{\beta_n + \beta_{n-1} + ... + \beta_j} < \frac{\alpha_n + \alpha_{n-1} + .. + \alpha_j + \alpha_\ell}{\beta_n + \beta_{n-1} + ... + \beta_j + \beta_\ell}, \tag{9}$$

which is a contradiction. Hence

$$\frac{\alpha_\ell}{\beta_\ell} \le \frac{\alpha_{\ell+1}}{\beta_{\ell+1}}, ..., \frac{\alpha_{n-1}}{\beta_{n-1}}, \frac{\alpha_n}{\beta_n}. \tag{10}$$

Now according to Vidal's work[31] if

$$P(|\psi\rangle \to |\phi\rangle) < P(|\phi\rangle \to |\psi\rangle),$$

then $\ln\frac{\beta_\ell}{\alpha_\ell}$ is positive. This implies that if the transformation $|\phi\rangle \to |\psi\rangle$ is more probable than the transformation $|\psi\rangle \to |\phi\rangle$, we have $A_\ell(\rho_\psi) > A_\ell(\rho_\phi)$. The converse is also true. Hence, the ℓ-th component of the potential difference $\triangle A_\ell$, which is the largest difference between the components of the state potentials, determines in which direction the transformation is more probable (see the following example). Consider three states $\psi_k \in C^4 \otimes C^4$, the square of the Schmidt coefficients of k-th state being $\vec{\alpha}_k$, where

$$\begin{aligned}
\vec{\alpha}_{k=1} &\equiv \frac{1}{122}(90, 12, 10, 10), \\
\vec{\alpha}_{k=2} &\equiv \frac{1}{122}(55, 55, 6, 6), \\
\vec{\alpha}_{k=3} &\equiv \frac{1}{122}(40, 40, 40, 2).
\end{aligned} \tag{11}$$

According to Vidal's theorem[31]

$$\begin{aligned}
P(\psi_1 \to \psi_2) &= \%32,\ P(\psi_2 \to \psi_1) = \%60, \\
P(\psi_1 \to \psi_3) &= \%39,\ P(\psi_3 \to \psi_1) = \%20, \\
P(\psi_2 \to \psi_3) &= \%28,\ P(\psi_3 \to \psi_2) = \%33.
\end{aligned} \tag{12}$$

Now let us use quantum affinity to predict the results above. The potential differences ΔA for these transformations read

$$\begin{aligned} A(\rho_{\psi_2}) - A(\rho_{\psi_1}) &= (0.49, -1.52, 0.51, 0.51), \\ A(\rho_{\psi_3}) - A(\rho_{\psi_1}) &= (0.81, -1.20, -1.38, 1.60), \\ A(\rho_{\psi_3}) - A(\rho_{\psi_2}) &= (0.31, 0.31, -1.89, 0.69). \end{aligned} \tag{13}$$

For transformations $\psi_1 \rightleftharpoons \psi_2$, since $\Delta A_\ell = -1.52$ then the transformation $\psi_2 \to \psi_1$ is more probable which completely agrees with the previous result. In the same way for transformations $\psi_1 \rightleftharpoons \psi_3$ and $\psi_2 \rightleftharpoons \psi_3$ we have $\Delta A_\ell = 1.60$ and $\Delta A_\ell = -1.89$, respectively. Thus transformations $\psi_1 \to \psi_3$ and $\psi_3 \to \psi_2$ are more probable.

4 Supplementary Note 4

The entries of the thermal Gibbs state can be approximated with arbitrarily high accuracy with rational numbers as[32]

$$\rho^\beta = (\frac{D_1}{D}, ..., \frac{D_d}{D}),\ D = \sum_{i=1}^{d} D_i,$$

where $D_i, D \in \mathbb{N}$. A d-dimensional probability distribution $\mathbf{p}$ is sent to a D-dimensional probability distribution $\hat{\mathbf{p}}$ by an embedding map Γ^β as follows[32]

$$\hat{\mathbf{p}} = \Gamma^\beta(\mathbf{p}) \equiv (\frac{p_1}{D_1}, ..., \frac{p_1}{D_1}, ..., \frac{p_d}{D_d}, ..., \frac{p_d}{D_d}).$$

Now consider two states ρ and σ block diagonal in energy eigenbasis with probability vectors $\mathbf{p}$ and $\mathbf{q}$, respectively. The necessary and sufficient conditions for block diagonal state interconversion under thermal operations is expressed as[32]

$$\mathcal{E}^\beta(\rho) = \sigma \ \ \textit{iff}\ \ \hat{\mathbf{p}} \succ \hat{\mathbf{q}}, \tag{14}$$

where

$$\mathcal{E}^\beta(\rho) = Tr_B[U(\rho \otimes \rho_B^\beta)U^\dagger],$$

with U satisfying $[U, H + H_B] = 0$, H Hamiltonian of the system and H_B being arbitrary. Now using quantum affinity A and Theorem **1** we can state the following theorem as:

Theorem 3 *A state ρ block diagonal in energy eigenbasis can be transformed with certainty into another block diagonal state σ by thermal operations if and only if*

$$\bar{A}(\hat{\alpha}) > \bar{A}(\hat{\beta}), \tag{15}$$

in which α,β are the probability vectors of the states ρ and σ, respectively.

5 Supplementary Note 5

Regardless of the physical details, the dynamics of open quantum systems can be roughly divided into two categories based on the memory effect of the reservoir[3]: Markovian and non-Markovian dynamics. Here we examine the behavior of $A(\rho_s(t))$ during Markovian and non-Markovian evolutions and show how it acts as the thermodynamic force driving the flow and backflow of information. Any local-in-time master equation, for a quantum system having a d-dimensional Hilbert space, can be written in the form[33], in the interaction picture,

$$\dot{\rho}_s = \sum_{k=1}^{d^2-1} \gamma_k(t)[L_k(t)\rho_s L_k^\dagger(t) - \frac{1}{2}\{L_k^\dagger(t)L_k(t), \rho_s\}], \tag{16}$$

where the $L_k(t)$ form an orthogonal basis set of traceless operators, i.e.,

$$tr[L_k(t)] = 0,\ tr[L_j^\dagger(t)L_k(t)] = \delta_{jk}. \tag{17}$$

The dynamics is Markovian if and only if all decoherence rates $\gamma_k(t)$ are positive and correspondingly, non-Markovian when one or more of $\gamma_k(t)$ are negative[33]. $A(\rho_s(t))$ is a function of the map Λ_t, thus it behaves differently during the flow and the backflow. In order to separate the contributions of the flow and the backflow in $\bar{A}(\rho_s(t))$ we take the time derivative of $\bar{A}(\rho_s(t))$

$$\frac{d\bar{A}(\rho_s(t))}{dt} = -tr\{\dot{\rho}_s(t)\rho_s^{-1}(t)\}. \tag{18}$$

If we define

$$\frac{d\bar{A}^k}{dt} \equiv -tr\{\gamma_k(t)[L_k(t)\rho_s L_k^\dagger(t) - \frac{1}{2}\{L_k^\dagger(t)L_k(t),\rho_s\}]\rho_s^{-1}\}, \tag{19}$$

Eq. (18) can now be written as

$$\frac{d\bar{A}(\rho_s(t))}{dt} = \sum_{k=1}^{d^2-1} \frac{d\bar{A}^k(\rho_s(t))}{dt}. \tag{20}$$

Now the contributions of the tendency for the flow and backflow of information can be clearly seen separately.

References

1. De Donder, T., Van Rysselberghe, P. *Affinity* (Stanford University Press: Menlo Park, CA 1936).
2. Kondepudi, D. & Prigogine, I. *Modern Thermodynamics* (New York: Wiley 1998).
3. Breuer, H. P. & Petruccione, F. *The theory of open quantum systems* (Oxford University Press, Oxford, 2002).
4. Nielsen, M. A. & Chuang, I. L. *Quantum Computation and Quantum Information* (Cambridge University Press, 2000).
5. De Groot, S. R. & Mazur, P. *Non-Equilibrium Thermodynamics* (New York: Dover, 1984).
6. Chen, Y. H., Shi, Z. C., Song, J. Xia, Y. & Zheng, S. B. Coherent control in quantum open systems: An approach for accelerating dissipation-based quantum state generation. *Phys. Rev. A* ***96****, 043853 (2017).*
7. Chruscinski, D. Kossakowski, D. & Pascazio, P. Long-time memory in non-Markovian evolutions. *Phys. Rev. A* ***81****, 032101 (2010).*
8. Nielsen, M. A. Conditions for a Class of Entanglement Transformations. *Phys. Rev. Lett.* ***83****, 436 (1999).*
9. Hatano, T. & Sasa, S. -i. Steady-State Thermodynamics of Langevin Systems. *Phys. Rev. Lett.* ***86****, 3463 (2001).*
10. Manzano, G., Galve, F., Zambrini, R. & Parrondo, J. M. R. Entropy production and thermodynamic power of the squeezed thermal reservoir. *Phys. Rev. E* ***93****, 052120 (2016).*
11. Ahmadi, B., Salimi, S. & A. S. Khorashad, *arXiv: 1809.00611v2 (2018).*
12. Leff, H. S. & Rex, A. F. *in Maxwell's Demon: Entropy, Information, Computing* (Princeton University Press, 1990).
13. Landauer, R. Irreversibility and Heat Generation in the Computing Process. *IBM J. Res. Dev.* ***5****, 183 (1961).*
14. Breuer, H. P., Laine, E. & Piilo, J. Measure for the Degree of Non-Markovian Behavior of Quantum Processes in Open Systems. *Phys. Rev. Lett.* ***103****, 210401 (2009).*
15. Hall, M. J. W. Complete positivity for time-dependent qubit master equations. *J. Phys. A* ***41****, 205302 (2008).*
16. Chruściński, D. & Maniscalco, S. Degree of Non-Markovianity of Quantum Evolution. *Phys. Rev. Lett.* ***112****, 120404 (2014).*
17. Vacchini, B. A classical appraisal of quantum definitions of non-Markovian dynamics. *J. Phys. B* ***45****, 154007 (2012).*
18. Streltsov, A., Adesso, G. & Plenio, M. B. Colloquium: Quantum coherence as a resource. *Rev. Mod. Phys.* ***89****, 041003 (2017).*
19. Lostaglio, M., Jennings, D. & Rudolph, T. Description of quantum coherence in thermodynamic processes requires constraints beyond free energy. *Nat. Commun.* ***6****, 6383 (2015)*.
20. Santos, J. P., Céleri, L. C., Landi, G. T. & Paternostro, M. The role of quantum coherence in non-equilibrium entropy production. *Nat. Quan. Inf.* ***5****, 23 (2019)*.
21. Francica, G., Goold, J. & Plastina, F. Role of coherence in the nonequilibrium thermodynamics of quantum systems. *Phys. Rev. E* ***99****, 042105 (2019).*
22. Oppenheim, J., Horodecki, M., Horodecki, P. & Horodecki, R. Thermodynamical Approach to Quantifying Quantum Correlations. *Phys. Rev. Lett.* ***89****, 180402 (2002).*
23. Lostaglio, M., Korzekwa, K., Jennings, D. & Rudolph, T. Quantum Coherence, Time-Translation Symmetry, and Thermodynamics. *Phys. Rev. X* ***5****, 021001 (2015).*
24. Åberg, J. Catalytic Coherence. *Phys. Rev. Lett.* ***113****, 150402 (2014).*

25. Ćwikliński, P., Studziński, M., Horodecki, M. & Oppenheim, J. Limitations on the Evolution of Quantum Coherences: Towards Fully Quantum Second Laws of Thermodynamics. *Phys. Rev. Lett.* ***115****, 210403 (2015)*.

26. Baumgratz, T., Cramer, M. & Plenio, M. B. Quantifying Coherence. *Phys. Rev. Lett.* ***113****, 140401 (2014)*.

27. Das, S., Khatri, S., Siopsis, G. & Wilde, M. M. Fundamental limits on quantum dynamics based on entropy change. *Journal of Mathematical Physics* ***59****, 012205 (2018)*.

28. Alicki, R. The quantum open system as a model of the heat engine. *J. Phys. A* ***12****, L103 (1979)*.

29. Deffner, S. & Lutz, E. Generalized Clausius Inequality for Nonequilibrium Quantum Processes. *Phys. Rev. Lett.* ***105****, 170402 (2010)*.

30. Bhatia, R. *Matrix analysis* (Springer Science, Business Media, 2013).

31. Vidal, G. Entanglement of Pure States for a Single Copy. *Phys. Rev. Lett.* ***83****, 1046 (1999)*.

32. Chubb, C. T., Tomamichel, M. & Korzekwa, K. Beyond the thermodynamic limit: finite-size corrections to state interconversion rates. *Quantum* ***2****, 108 (2018)*.

33. Hall, M. J. W., Cresser, J. D., Li L. & Andersson, E. Canonical form of master equations and characterization of non-Markovianity. *Phys. Rev. A* ***89****, 042120 (2014)*.
